# Close Times

**Published:** 1892-05-21

**Authors:** 


### The Hospital

AN INSTITUTIONAL JOURNAL OF

Science, Medicine, Nursing, and Philanthropy.

*For Hospitals, Asylums, Infirmaries, Dispensaries, Medical Practitioners, Students, Nurses, and the Charitable Public.*

VOL. XII.—No. 295.] [MAY 21, 1892.

### CLOSE TIMES.

MR. A. J. BALFOUR hit upon a truth which had universality in it when, at the Newspaper Press Fund dinner last week, he suggested a "close time" for politicians. One of the last distinctions which the leader of the House of Commons would claim for himself would be that of scientific specialist; but that he is in true essence a man of science no sympathetic reader of his speeches can for a moment doubt. Every man of competent intelligence in these times strives to base his purposes and his actions upon great principles. This is one of the fruits of modern culture, and men act so almost unconsciously. But they also act after this fashion for reasons of high expediency; they wish to gain a reputation for judgment, for thoroughness, for trustworthiness, for consistency. They desire also to avoid the interruption and irritation of being pulled up by incompetent critics. A reputation of this kind takes a great deal of making, but when it is made it is generally the parent of enduring fortune and fame.

Who has made the discovery, it may be asked, that "close times" are universally serviceable? Nature has made it. The world in England is just waking up from the prolonged close time of a stern winter. The exhilarating energy and surpassing beauty of our English forests and fields at the present moment are standing concrete arguments in favour of close times, by which the most sceptical cannot fail to be convinced. We have a spring time beautiful with abounding vitality because we have had by way of preparation a five months' period of wintry death. Nature turned the key upon her storehouses of life last October, and kept living forms at the lowest ebb of vitality until the end of March. Then she set free her boundless resources, and miracle upon miracle has been the magnificent result. Winter is but one of nature's examples of close times. The birds are amongst the most familiar of close time observers. The wild animals, as well as those that are domesticated, live by the same rules. Men themselves are conscious of ebbings and flowings, and each of us feels in his own mind that an occasional close time is like a spring resurrection to exhausted body and mind.

Our object in seizing upon Mr. Balfour's passing thought is to insist upon the claim that scientific things and scientific methods of looking at things have upon the competent intelligence. Science is undoubtedly to be one of the guiding torches of the future. She has come to stay, as the Americans have it; and she has come to make herself felt. Those gentlemen who were sitting at the table with Mr. Balfour at the Newspaper Press Fund Dinner, and who applauded his suggestion of a "close time" for politics, did not, possibly, at the moment think how altogether admirable a thing it would be if they could enact a close time for themselves. If science has a great part to play in the future, literature has as great, or even a greater, task to complete. The literature of the daily and of the weekly press is not becoming less practically significant year by year, but more. What forces itself upon the consciousness is this, that, if the English world is to meet the demands of the future as the Englishmen of the past have always met the demands made upon them, the thinkers, the teachers, and the writers of the English-speaking race must be strong in judgment, high in courage, and of an intelligence both deep and wide.

No writing man can be strong in judgment, high in courage, and of an intelligence deep and wide, who works with his pen and with his brain without intermission from January till the last day in December. He may produce; but what he produces must inevitably be as tame as the paces of the canal-horse, whose only movement is a wooden walk from the beginning to the end of the year. Play and fallow were familiar ideas to grown men and agriculturists thirty yearty years ago. Now they are both to a large extent unknown. It is a thing which must have struck many people, as well as the writer, that nobody seems now to expect great books, great poems, great newspaper articles. Everywhere it is taken for granted that these things are not likely to be done. Nor will they be done if writers and thinkers persist in working without cessation, and in regarding as unnecessary long periods of breathing time, and weeks or months of fallow cultivation.

The spirit of science, the spirit of patriotism desires above all things to see great and heroic men, who can do great deeds, and play great parts in the world; men whom other men can and will follow. But commonplace, even though it be dutiful and worthy commonplace, never does great things. Great things require great efforts, and great preparation beforehand. Nature herself seems to prepare for prodigious efforts in the production of her striking results. What mad and mighty winds are unchained when the kingly forest trees are broken asunder or torn up by the roots! The sea never rises to overwhelming storms without a preparation of portents and marshalled thunder-clouds. There must ever be the period of gestation before the day of birth. Even in the merely mechanical world the powerful engine yoked to the heavy train must often make backward revolutions before it can plunge forward into the darkness with its sullen and massive load.

Mr. Balfour sees the British Parliament with eyes of science and reason. It is a Parliament that works by wasteful methods. When it is in session it squanders the day and works in the night; when it is on holiday it puts on all its emotional steam to convince those that are already convinced, and to excite jaded minds that have been worn out with a plethora of previous excitement. There is probably no hope that the older men will mend; but if Mr. Balfour may be taken as a type of the more scientifically cultured younger men, we shall see better things before we are much older. Parliamentary men will work as if they were men of sense; they will work in the day, and the night they will devote to sleep. They will then have serviceable brains, and not merely excitable nervous systems that have no other activity but the reflex. And when the period of their session is over, they will not tear up and down the country on speech-making tours of worry that knows no cessation: they will rest; they will enjoy themselves; they will give nature time and opportunity to soothe, to refresh, to restore, to inspire, to make them young and lusty as Shakespeare's men were, and to fit them to play the part of thinkers in the world, and not of mere sensitive telephones.

Stolidity is a great endowment when it is allied with intelligence. To see mad passions raging all around, and to sit still unmoved and immovable! To have your own set purposes maturing in your brain, and to carry these out slowly but irresistibly, like the steadfast roll of the marching waves that swallow up the unresisting shore. This is the kind of thing that is wanted in states whose people consist of tens of millions, and whose contending factions are numbered by the thousand. This is what the student of nature is constantly impressed by: the steadiness of the world; the ordered progression of seasons and events; the gentle ebb and the mighty flow; the period of prolonged rest, followed by the irresistible effort which makes an old world new every succeeding spring.

The people of this generation have had enough of excitement. They have drunk to the fill of the idea of the struggle for survival. A rational science demands now that they discontinue that particular lesson of nature's school, and turn their attention to another. Mental rest, mental sleep, repose, patience, a purpose that has confidence in itself and in the destiny of the world—these are the things which science tells us ought to stand over against struggles and fierce fightings, and to counter-balance them. By way of immediate application of such generalisations, one would say that the long days of summer are close upon us all, and that they invite to a period of enjoyment. It grieves the physiologist to see the jaded and anxious men which pour along our streets and fill the railway trains that converge upon the great cities of our Empire. Those men have been the makers of the modern world; they are still the Atlases upon whose shoulders it is firmly poised. Alas! that the only reward for their strenuous work should be feeble health and deepening melancholy. "A close time" from all this work, and still worse worry, is what the physiologist insists upon. He who thinks he cannot afford such a "close time," is the very man who should give himself a rest of sufficient length, and that at the earliest possible date.

